# Editorial on the Special Issue “Image and Video Processing for Blind and Visually Impaired”

**DOI:** 10.3390/jimaging11120430

**Published:** 2025-12-03

**Authors:** Zhigang Zhu, John-Ross Rizzo, Hao Tang

**Affiliations:** 1 Department of Computer Science, The City College, The City University of New York, New York, NY 10031, USA; 2 Department of Computer Science, The Graduate Center, The City University of New York, New York, NY 10016, USA; htang@bmcc.cuny.edu; 3 Department of Rehabilitation Medicine and Department of Neurology, NYU Langone Health, New York, NY 10016, USA; johnrossrizzo@gmail.com; 4 Department of Mechanical and Aerospace Engineering, NYU Tandon School of Engineering, New York, NY 11201, USA; 5 Department of Biomedical Engineering, NYU Tandon School of Engineering, New York, NY 11201, USA; 6 Department of Computer Information Systems, Borough of Manhattan Community College, The City University of New York, New York, NY 10007, USA

Over 2.2 billion people across the world live with vision loss [1,2]. Vision plays a primary role in the efficient capture and integration of sensory information from the surrounding environment, and is critically involved in the complex process of sensory transduction through higher level cortical interpretation, enabling the localization and recognition of spatial layouts and objects; the comprehension of the three-dimensional relationships among objects and spatial geometry, including egocentric perspective or one’s own location relative to landmarks; and, on a meta-level, spatial cognition. Visual impairment encompasses a broad spectrum of vision loss, categorized by severity and underlying causes [3,4,5]. Common types include *refractive errors* (nearsightedness, farsightedness, or astigmatism), which cause blurry vision; different types of *color blindness*, characterized by color vision deficiency; *cataracts*, characterized by the clouding of the eye’s lens; *glaucoma*, optic nerve damage that affects peripheral vision; *diabetic retinopathy*, due to damaged blood vessels from diabetes; and *age-related macular degeneration* (*AMD*), which impacts central vision. Virtually all aspects of life are affected by a loss of visual input and various types of visual impairment. More broadly, visual impairment leads to difficulties in performing activities of daily living, affects safe mobility, decreases social participation, prevents individuals from accessing rich digital media, and results in diminished independence and quality of life [6]. Besides the immediate limitations caused by sensory loss, physical/environmental infrastructure (e.g., a lack of accessibility) and social factors (e.g., discrimination and a lack of educational resources) amplify visual impairment-related limitations and restrictions.

*This Special Issue on Image and Video Processing for Blind and Visually Impaired*, in the *Journal of Imaging*, was proposed to report on the solutions to these challenges for the community. For this Special Issue, we sought original contributions that comprise innovative methods and applications, in particular those using image and video processing, which can be used to promote independence and community living among people of all ages with low vision, blindness (including color blindness), and other visual impairments. For consistency, we refer to all types of visual impairment as ***blind and visually impaired (BVI)***, and “individuals who are blind or visually impaired”, or simply “BVI individuals (users)”, will be used throughout this Editorial where appropriate. However, when the topics concern only people who are blind or have low vision (BLV), we use “BLV individuals” or “BLV users”. For this Special Issue, the topics we identified include, but are not limited to, the following:

1. Increased *access* to graphical information, signage, and travel information for BVI users, both in digital media format or in physical spaces, or devices and appliances with digital displays and control panels produced through AI-based image and video processing.
2. Improved non-visual or enhanced visual *orientation and mobility guidance* for users who are blind or have low vision in both indoor and outdoor environments by using portable and/or mobile image and video processing.
3. The increased *participation of BVI individuals* in science, technology, engineering, arts, mathematics, and medicine (STEAM2) education and careers through the use of augmented reality and assistive technology techniques with image and video processing.

All submissions that passed pre-check were peer-reviewed through a single-blind peer review process. In the end, eight papers have been accepted to this Special Issue, and they have been published continuously in the journal (as soon as they were accepted) and listed together on the Special Issue website (https://www.mdpi.com/journal/jimaging/special_issues/3917K65834, accessed on 24 November 2025). This Editorial serves to provide a brief overview of this Special Issue. In particular, this Editorial discusses how this Special Issue addresses users’ needs and the knowledge gaps mentioned above, and what future research should be considered. In the following discussions, we gave a short name to each piece of work and put them in the aforementioned three categories of work.

## 1. Digital Access

In terms of *digital and physical access* for people who have visual impairments, all of the eight papers offer solutions that address various issues. In particular, five papers stand out for their original solutions to unique problems across digital access, highlighting obstacles across varied online experiences, from encountering misleading ads to exploring art and retrieving information.

***Deceptive Ad Detection***. The first paper, *Detecting Deceptive Dark-Pattern Web Advertisements for Blind Screen-Reader Users* [C8], develops an algorithm to identify contextually deceptive ads that mislead screen-reader users. Prior research on non-visual web interaction rarely examined how ads designed for sighted users affect blind screen-reader users, leaving them vulnerable to contextually deceptive advertisements that blend into surrounding content. To fill this gap, the authors of this paper built a detection model that leverages a multi-modal combination of handcrafted and automatically extracted features to determine if a particular ad is contextually deceptive. Evaluations of this model on a representative test dataset and “in-the-wild” random websites yielded F1 scores of 0.86 and 0.88, respectively.

***Art Image Captioning***. While the first study tackles misleading web advertisements that disrupt non-visual browsing, the next paper, *Images, Words, and Imagination: Accessible Descriptions to Support Blind and Low Vision Art Exploration and Engagement* [C7], shifts from commercial web content to cultural experiences, exploring how to deliver rich and trustworthy art descriptions for BVI museum visitors. To address this challenge, the team of this paper conducted two studies: (1) a qualitative study asking BLV participants (11 in total) for their preferences for layered description characteristics; (2) an evaluation of several current models for image captioning as applied to an artwork-based image dataset. Recommendations are provided for researchers working on accessible image captioning and museum engagement applications through a focus on spatial information access strategies. The paper also discussed the limitations of the work, such as the ethical implications of how artwork datasets are acquired to train and test an image captioning model, and the use of the newer generative models, including ChatGPT4 for generating artwork captions.

***Visual-Related Help-Seeking***. Extending the focus from static art interpretation to everyday digital information retrieval, the third study, *Help-Seeking Situations Related to Visual Interactions on Mobile Platforms and Recommended Designs for Blind and Visually Impaired Users* [C4], investigates how BVI users seek help when interacting with mobile digital libraries and identifies concrete design strategies to reduce those barriers. The authors of this paper conducted a large-scale study with 120 BVI users searching for information in six digital libraries (DLs) on four types of mobile devices, using questionnaires, think-aloud protocols, transaction logs, and interviews. They identified seven types of help-seeking difficulties. Based on these findings, they recommended design improvements, including meaningful icon labels, intuitive video descriptions, structured pagination cues, the clearer separation of titles from thumbnails, AI-based image/graph recognition, and limiting screen-reader interactions to active windows. The paper also found limitations in generalizing results across different DL types and in using virtual data collection, guiding future design and evaluation efforts.

The following two papers deal with color blindness. Color vision deficiency (CVD) affects hundreds of millions worldwide, creating a need for Daltonization techniques that improve detail visibility without distorting natural image appearance.

***Trichromat-Friendly Daltonization***. Daltonization is a popular approach to remap confusing colors to more distinguishable hues so people with CVD can perceive visual information more accurately. However, it often distorts the natural appearance of images. In *Leveraging Achromatic Component for Trichromat-Friendly Daltonization* [C2], the authors introduced a novel Daltonization method that modifies only the achromatic component of images to preserve image naturalness while enhancing detail visibility for individuals with CVD. Compared with the anisotropic Daltonization baseline, the proposed method was preferred by over 90% of CVD participants and 95% of trichromats for its more natural appearance, while achieving comparable detail discrimination despite slightly lower objective contrast scores (65% contrast improvement for protan cases versus 70% for the baseline).

***Accessible Web Color Schemes***. Together with advances in image Daltonization, a complementary study broadened accessibility efforts from enhancing individual images to ensuring entire web interfaces are visually inclusive. In *Research on the Accessibility of Different Colour Schemes for Web Resources for People with Colour Blindness* [C1], the authors surveyed people with different types of CVD and calculated color deviation values to objectively assess how different color groups are perceived. Based on these findings, they provided concrete recommendations for selecting optimal website color schemes to improve comfort and accessibility for the broadest audience. The study emphasizes the need to consider users with color vision impairments and offers practical guidelines for selecting website color schemes to improve accessibility and effectiveness.

Shifting from digital inclusion to physical accessibility, the next set of papers [C3, C5, C6] moves beyond improving online interaction to addressing how BVI individuals perceive, navigate, and engage with the built environment, which is related to *orientation and mobility guidance*.

## 2. Orientation and Mobility Guidance

This group of papers tackles accessibility in physical space from three different angles: infrastructure inventory, environmental interaction, and spatial awareness.

***Pedestrian-Accessible Infrastructure Inventory***. Accurate, comprehensive inventories of pedestrian infrastructure, especially accessibility-critical street furniture, are limited, hindering effective planning for people with BVI. In *Pedestrian-Accessible Infrastructure Inventory: Enabling and Assessing Zero-Shot Segmentation on Multi-Mode Geospatial Data for All Pedestrian Types* [C6], the team of this paper presented a Segment Anything Model (SAM)-based pedestrian infrastructure segmentation workflow, which focuses on mobile LiDAR point cloud street-view image data and satellite imagery data to create scalable, zero-shot segmentations of pedestrian infrastructure. The study defines an expanded inventory that includes street furniture elements often overlooked in traditional maps. It details how to prepare and represent multi-mode data for the SAM and shows that this approach reliably maps pedestrian assets. This method provides a scalable tool for GIS professionals, city managers, and transportation agencies who design and manage accessible infrastructure, as well as for BVI walkers who rely on these services.

***Foundation Model for BLV Interaction***. Building on the city-scale mapping of pedestrian assets, the next study, *A Multi-Modal Foundation Model to Assist People with Blindness and Low Vision in Environmental Interaction* [C5], shifts from mapping the environment to interpreting it in real time, focusing on how BVI individuals can perceive dynamic scenes and avoid hazards. Existing assistive tools often lack the robustness to handle dynamic environments, the team of this work presents a multi-modal foundation model framework for real-time environmental understanding. The method begins by leveraging a large image tagging model (i.e., Recognize Anything Model (RAM)) to identify all common objects present in the captured images. The recognition results and user queries are then integrated into a prompt, tailored specifically for BLV users, using prompt engineering. By combining the user prompt and input image, a vision–language foundation model (i.e., InstructBLIP) generates detailed and comprehensive environmental descriptions and identifies potential risks by analyzing objects and scenic landmarks relevant to the prompt. The proposed approach is evaluated on both indoor and outdoor datasets, demonstrating its ability to recognize objects accurately and provide insightful environmental descriptions and analyses of the for pBLV.

***Visual Impairment Spatial Awareness***. After demonstrating how foundation models can describe and interpret outdoor and unfamiliar spaces, the next paper, *Visual Impairment Spatial Awareness System for Indoor Navigation and Daily Activities* [C3], focuses on structured indoor navigation, integrating multiple sensing layers to give visually impaired users fine-grained spatial awareness. The authors of this paper introduced the Visual Impairment Spatial Awareness (VISA) system, designed to holistically assist visually impaired users in indoor activities through a structured, multi-level approach. At the foundational level, the system employs augmented reality (AR) markers for indoor positioning, neural network models for advanced object detection and tracking, and depth estimation methods for object localization. At the intermediate level, it supports obstacle avoidance and pathfinding. At the advanced level, it synthesizes these capabilities to enable users to navigate complex environments and locate specific items. The VISA system exhibits an efficient human–machine interface (HMI), incorporating text-to-speech and speech-to-text technologies. Evaluations in simulated real-world environments confirm that the VISA system efficiently assists visually impaired users in indoor navigation, object detection and localization, and label and text recognition.

## 3. BLV Participation in Education and Careers

Increasing the *participation of BVI individuals* in science, technology, engineering, arts, mathematics, and medicine (STEAM2) education and careers through the use of augmented reality and assistive technology techniques with image and video processing will have a significant societal and economic impact. Even though we do not have specific papers on this category in this Special Issue, the discussed ideas, proposed methods, and implemented evaluations of all papers touched on this topic. We will briefly detail the proposed approaches/ideas in these papers for serving this purpose across three application domains: STEAM2 education, career training, and workplace assistance.

***STEAM2 Education***. Educational materials are not fully accessible to BVI people. This is especially true for multimedia materials, mathematical documents, and arts and paintings. In addition, K-12 and college education use more and more online materials, which increases the digital divide between sighted and BVI learners. This collection of papers shines a light on some traditionally underexplored topics. Two pieces of work—*Art Image Captioning* [C7] and *Foundation Model for BLV Interaction* [C5]—indicated that deep learning vision models, especially vision–language models (VLMs), can be a tremendously helpful tool for translating images of art, designs, mathematics, etc., into accessible text and audio descriptions and providing an interactive mechanism for BVI learners to delve into more specific information with effective follow-up prompts. *Deceptive Ads Detection* [C8] and *Visual-Related Help-Seeking* [C4] could provide powerful tools for BVI learners to explore online multimedia materials and digital libraries with few hurdles. Finally, *Trichromat-Friendly Daltonization* [C2] and *Accessible Web Color Schemes* [C1] are geared towards a better visual experience for learners who have color vision deficiency and color blindness. Inspired by these works, future research should move beyond individual solutions toward an integrated ecosystem of AI-driven educational tools. Such a framework could dynamically adapt to different types of visual impairment and specific learning tasks such as reading text; interpreting images, videos, diagrams, paintings, and math equations; and exploring rich multimedia materials in different formats (PDF, HTML, metaverse, etc.).

***Life-long Career Training***. Life-long career training implies that BVI individuals, who may be in the workforce or not, can learn new skills and knowledge on their own schedules to keep pace with the ever-changing landscape of job opportunities. This is especially true in the age of AI. The thoughts we have regarding *STEAM2 Education* can also be applied here, and probably even more so, since those AI-based methods can be used as chatbots to help BVI users learn and adapt to new concepts at their own pace. The AI tools can also be customized to each user’s own preferences. Therefore, discussions on customized tools for specific tasks are also valid here. Future research needs to consider the accessibility of these AI tools, and how they can meet the individual needs of BVI users without increasing their cognitive loads, with a particular focus on developing accessible interfaces for using vision–language models and conducting large-scale user studies investigating the use of these tools for online materials, such as digital libraries.

***Workplace and Daily Assistance***. In addition to the AI tools focused on digital media access, as discussed above, workplace and daily assistance tools that help people navigate cluttered environments are also highly needed. This is especially true for workers involved in commuting and dealing with physical entities. The work on *Pedestrian-Accessible Infrastructure Inventory* [C6] is actually a good first step in building accessible infrastructure for pedestrians who are visually impaired, among other disabilities, to access transportation stations, workplaces, and daily errands. The work on *Visual Impairment Spatial Awareness* [C3] addresses the issues of navigation and situation awareness for BVI travelers so that they can live an independent life and have more freedom in daily life and the workplace, using the robotics and computer vision technologies researchers have developed over recent decades. Finally, the work on *Foundation Model for BLV Interaction* [C5] demonstrates that vision–language models (VLMs) can provide BVI users with accurate and detailed higher level understanding of surrounding environments, showing the promise of AI technology for allowing BVI users to live and work at a level of freedom that we have never imagined before. However, making these tools a part of daily assistance still requires more studies on their accuracy, robustness, and real-time performance.

## 4. Future Directions

In conclusion, we hope that this Special Issue will inspire more researchers and developers in computer vision and AI to consider the needs of people who are blind or visually impaired. As scientists are increasingly implementing deep learning, foundation models, and artificial intelligence into their work [7,8,9,10], it is imperative that they pause and think about the accessibility (both digital and physical), ethics (including privacy) and usability (including multimodal smart interfaces) of each and every technological development [11,12,13,14,15,16,17,18,19]. In support of such a goal, we must conduct and advocate for more user studies that focus AI-based tools on the populations of greatest need, otherwise we run the risk of an unchecked, ever-expanding AI divide [18,19].
